# Effects of risk factor-based targeted nursing intervention on psychological status, sleep quality, and pain in patients with trigeminal neuralgia

**DOI:** 10.3389/fneur.2025.1681364

**Published:** 2025-12-11

**Authors:** Jing Zhang, Yao Wang, Tao Ding, Xue Jiang, Huayu Chen

**Affiliations:** Department of Neurosurgery, Jiangsu Provincial People’s Hospital, The First Affiliated Hospital with Nanjing Medical University, Nanjing, China

**Keywords:** trigeminal neuralgia, pain perception, anxiety, depression, Sleep quality, individualized nursing

## Abstract

**Background:**

To identify clinical factors associated with pain perception, psychological status, and sleep quality in patients with trigeminal neuralgia (TN) and to evaluate the clinical efficacy of individualized comprehensive nursing interventions in alleviating pain, anxiety, depression, and sleep disorders.

**Methods:**

This study combined retrospective with prospective design. The retrospective analysis included 162 patients with TN admitted to our hospital from January 2020 and March 2022. Patients were grouped based on their scores on the Visual Analogue Scale (VAS), Hospital Anxiety and Depression Scale (HADS), and Pittsburgh Sleep Quality Index (PSQI). In the prospective arm, 64 eligible patients were assigned into intervention (individualized nursing care) and control (standard routine care) groups using a random number table. Changes in VAS, HADS, and PSQI scores before and after the intervention were compared, along with assessments of nursing adherence and patient satisfaction.

**Results:**

Retrospective analysis revealed that sex, frequency of pain episodes, comorbid hypertension, and history of surgical treatment were associated with pain scores. Pain distribution and previous treatment methods were correlated with anxiety/depression. Frequency of pain and pain distribution were major factors affecting sleep quality. In the prospective study, the intervention group showed significantly lower VAS, HADS-Anxiety, HADS-Depression, and PSQI scores. The intervention group also demonstrated better adherence to nursing and higher nursing satisfaction.

**Conclusion:**

Risk-factor based targeted nursing interventions can alleviate pain, negative emotions, and sleep disturbances, while enhancing adherence and patient satisfaction, demonstrating strong clinical value in TN management.

## 1 Introduction

Trigeminal neuralgia (TN) is a common neuropathic pain syndrome characterized by sudden, severe, paroxysmal facial pain (1), primarily caused by damage or dysfunction of the branches of the trigeminal nerve. It profoundly affects patients’ quality of life and mental health (2). Clinically, TN often presents as sharp, electric shock-like or stabbing pain (3), which is typically brief but occurs frequently. In some cases, the condition progresses into persistent, intractable pain that poses significant therapeutic challenges (4). Although pharmacological treatments, such as carbamazepine and oxcarbazepine, are effective in most cases (5), long-term use may lead to drug resistance, hepatotoxicity, nephrotoxicity, and cognitive side effects. Minimally invasive procedures, including Gamma Knife radiosurgery and radiofrequency ablation, show efficacy in some refractory TN patients but carry risks of recurrence and postoperative complications (6). Therefore, effective pain management, psychological support, and improvements in quality of life are critical components of comprehensive TN care.

In recent years, growing recognition of the role of psychosocial factors (7) in the pathophysiology of chronic pain has led to increased clinical attention to the high prevalence of anxiety, depression, and sleep disorders in patients with TN (8). Studies have shown that the incidence of anxiety and depression in TN patients can reach as high as 30%–60% (9), while sleep disturbances are commonly observed among individuals experiencing moderate to severe pain. There is a bidirectional relationship between pain and emotional states (10) and between pain and sleep quality (11). Persistent pain can trigger emotional dysregulation, which in turn lowers pain tolerance, creating a vicious cycle. However, most existing research focuses on pharmacologic or surgical interventions for TN, and relatively few studies have systematically examined the interrelations among pain characteristics, psychological status, and sleep quality, particularly from an integrated nursing perspective that emphasizes holistic patient care.

Moreover, although the concept of individualized nursing has been gradually applied in the management of various chronic conditions, its implementation in TN remains in the exploratory phase. Developing a multidimensional and comprehensive nursing intervention tailored to the pain characteristics, emotional fluctuations, and sleep disorders in TN patients, and scientifically evaluating its efficacy, remains a pressing issue in clinical nursing research.

Therefore, this study was conducted in two stages: a retrospective phase to systematically analyze the potential risk factors affecting pain, psychological status, and sleep quality in TN patients and to clarify the underlying mechanisms; and a prospective randomized controlled phase to assess the effectiveness of individualized comprehensive nursing interventions in improving pain severity, emotional status, and sleep quality. The ultimate aim is to explore the feasibility and efficacy of clinical feature-based nursing strategies in TN patients. The findings are expected to provide a full-cycle, personalized nursing support framework to enhance patients’ overall rehabilitation quality and to enrich both the theoretical and practical foundations of pain management in nursing care.

## 2 Methods

### 2.1 Study design and participants

This study adopted a mixed-method design incorporating both retrospective and prospective approaches, aiming to explore factors influencing pain perception, psychological status, and sleep quality in patients with TN, and to further evaluate the clinical efficacy of individualized comprehensive nursing interventions. In the retrospective phase, clinical data from 162 patients diagnosed with TN and treated at our hospital between January 2020 and March 2022 were collected and analyzed. In the prospective phase, 64 hospitalized patients meeting the inclusion criteria were recruited between April 2022 and March 2024 and randomly assigned to an intervention group (*n* = 32) or a control group (*n* = 32) using a random number table. The intervention program was implemented and verified accordingly. This study was approved by the institutional ethics committee of Jiangsu Provincial People’s Hospital, The First Affiliated Hospital with Nanjing Medical University (approval number: 2022-SR-181). Informed consent was waived for the retrospective phase, while written informed consent was obtained from all participants in the prospective phase.

To enhance methodological rigor and ensure replicability, several additional procedures were implemented in both the retrospective and prospective phases. In the retrospective analysis, potential confounding factors—including medication type and dosage, history of psychiatric illness, and other comorbid conditions—were systematically reviewed. Patients receiving ongoing psychiatric treatment or with documented psychiatric diagnoses (e.g., major depressive disorder, generalized anxiety disorder) were excluded to reduce the influence of pre-existing mental illness on psychological and sleep assessments. Medication records were screened and any substantial between-group differences were treated as covariates during statistical analysis.

In the prospective randomized controlled trial, randomization was performed using a computer-generated random number table, and allocation concealment was ensured through the use of sealed, opaque, sequentially numbered envelopes prepared by an independent researcher not involved in patient care or outcome assessment. All outcome assessors were blinded to group assignments to minimize measurement bias. Sample size was determined based on an *a priori* power analysis; although the final sample size was modest, 95% confidence intervals for all primary outcomes (VAS, HADS-A/D, and PSQI) were calculated to improve statistical transparency.

The individualized comprehensive nursing intervention was delivered by nurses certified in psychological counseling and trained in standardized behavioral protocols. Psychological support included structured supportive counseling, relaxation training, and music-assisted interventions, administered 2–3 times per week for 20–30 min per session. The pain-management component followed national TN care guidelines and incorporated non-pharmacological strategies (e.g., localized heat therapy, facial relaxation exercises), while pharmacological regimens were kept consistent between groups. Adherence to non-pharmacological practices was monitored through daily patient logs and weekly follow-up checks by nursing staff. Taken together, these measures strengthened the internal validity of the study and ensured the reproducibility of the intervention protocols.

To address potential concerns regarding statistical power in the prospective phase (*n* = 64), we conducted an *a priori* power analysis targeting medium effect sizes, which confirmed that the sample size was sufficient for detecting clinically relevant changes in the primary outcomes. Nevertheless, given the multidimensional nature of the assessed domains (pain intensity, anxiety, depression, and sleep quality), we additionally reported 95% confidence intervals (CI) for all primary outcome measures to strengthen statistical transparency and enhance interpretability. The inclusion of confidence intervals allows readers to evaluate the precision and robustness of the observed effects, even in the context of a modest sample size.

### 2.2 Sample size calculation and effect size analysis

To ensure adequate statistical power for the prospective randomized controlled trial, the sample size was estimated based on changes in anxiety scores as measured by the Hospital Anxiety and Depression Scale-Anxiety subscale (HADS-A), the primary endpoint. Referring to previous literature and preliminary data from our hospital, a mean reduction of 2.5 points in HADS-A scores was expected in the intervention group (standard deviation: 2.0), compared to a 1.0-point reduction in the control group (standard deviation: 2.0). Assuming a two-sided significance level (*α*) of 0.05 and a power (1–*β*) of 0.80, the minimum required sample size per group was calculated to be 28. Allowing for an anticipated 10% dropout rate, 32 participants were ultimately enrolled in each group, yielding a total sample size of 64 and ensuring sufficient representativeness and robustness.

The retrospective dataset of 162 TN patients met the basic sample size requirements for risk factor analysis. To further evaluate the strength of associations and intervention effects, effect size metrics were applied (12, 13). Differences in continuous variables were assessed using Cohen’s d, while associations between categorical variables were measured using Cramér’s V. The intervention group showed a Cohen’s d of approximately 1.0 for Visual Analogue Scale (VAS) scores, 0.80 for HADS-A, and 0.75 for HADS-Depression (HADS-D), indicating medium to large effect sizes and demonstrating significant improvements in pain and emotional states. For categorical variables, Cramér’s V between sex and pain perception was approximately 0.17 (weak effect), while the value for pain distribution (e.g., involvement of multiple branches) and anxiety was approximately 0.43 (moderate effect), supporting a correlation between pain burden and emotional distress. These results collectively affirm that the study design and conclusions are statistically well-supported and interpretable.

### 2.3 Inclusion and exclusion criteria

Inclusion criteria were as follows: (1) age ≥18 years; (2) diagnosis of TN in accordance with the International Headache Society criteria (14); (3) ability to self-report and communicate verbally; (4) complete clinical records and ability to complete questionnaires.

Exclusion criteria included: (1) coexisting severe psychiatric or organic neurological disorders (e.g., stroke, brain tumor); (2) presence of other chronic pain conditions or severe systemic diseases; (3) communication or cognitive impairments; and (4) incomplete data or withdrawal during follow-up.

### 2.4 Grouping and nursing interventions

In the retrospective phase, patients were categorized based on their pain, psychological, and sleep assessment scores. Pain status was assessed using the VAS (15), and patients were divided into a “low-VAS” group (VAS = 0) and a “high-VAS” group (VAS > 0). Psychological status was evaluated using the HADS (16). Patients with a HADS-A or HADS-D score ≥8 were classified as being at risk for anxiety or depression, while others served as controls. Sleep quality was assessed with the PSQI (17). A total score >5 indicated poor sleep quality, and ≤5 indicated good sleep quality.

In the prospective randomized controlled phase, all participants received a 4-week nursing intervention. Based on random number table method, the control group received routine nursing care, including disease education, medication guidance, and rehabilitation advice. The intervention group received individualized comprehensive nursing interventions in addition to routine care. These interventions were designed based on risk factors identified in the retrospective phase and encompassed three main components: (1) Psychological intervention, including HADS screening, structured psychological interviews, counseling for anxiety/depression, music-assisted relaxation training, and family/social support; (2) Sleep intervention, involving PSQI assessment, sleep hygiene education, bedtime relaxation techniques (e.g., progressive muscle relaxation and aromatherapy), and nighttime pain management adjustments; and (3) Pain management, including a personalized analgesia plan (medications + physical therapy), identification and avoidance of pain triggers, pain diary keeping, and adherence education. All interventions were delivered by trained nursing staff twice per week over four consecutive weeks.

#### 2.4.1 Individualized comprehensive nursing intervention

The individualized comprehensive nursing intervention consisted of three structured components—psychological support, pain management, and sleep-focused behavioral guidance—delivered according to a standardized protocol to ensure reproducibility. All interventions were administered by senior nurses with more than 5 years of neurology or pain-management experience and additional training in supportive psychotherapy skills.

Psychological support was provided twice weekly in 20–30-min one-on-one sessions. The approach followed a supportive and psychoeducational framework, supplemented by basic cognitive–behavioral techniques such as cognitive reframing, guided breathing, and progressive relaxation. Patients were encouraged to express emotions, clarify illness-related misconceptions, and strengthen adaptive coping strategies. Music-assisted relaxation (15–20 min of low-tempo instrumental music) was offered as an auxiliary method to reduce anxiety and enhance relaxation.

Pain management followed a structured pathway integrating pharmacologic and nonpharmacologic measures. Pharmacologic therapy—including carbamazepine, oxcarbazepine, or gabapentin—was administered strictly according to physician prescriptions. Nurses monitored medication adherence through daily pill counting and brief self-reports, documented adverse reactions, and ensured communication of necessary dose adjustments. Nonpharmacologic measures included localized heat application (10–15 min daily), acupressure using standardized anatomical points, jaw relaxation exercises, and individualized training to avoid known triggers such as chewing habits, abrupt temperature changes, or certain foods. All techniques were demonstrated during initial sessions and reinforced throughout the intervention period.

Sleep-quality enhancement involved structured sleep-hygiene education and practical behavioral guidance. Patients received instructions on establishing consistent sleep–wake schedules, reducing evening stimulation (e.g., caffeine), improving sleep environment (light, sound, and temperature control), and practicing brief relaxation or breathing exercises before bedtime. Daily checklists were used to monitor adherence to sleep-hygiene recommendations.

All interventions were delivered over a four-week period. Nursing activities and adherence monitoring were documented in individualized intervention logs. Importantly, outcome assessments were conducted by independent evaluators blinded to group allocation to minimize measurement bias. The standardized and multi-component nature of this protocol ensured consistency across participants while still allowing tailoring based on individual symptom profiles.

### 2.5 Assessment tools and outcome measures

The study employed several validated instruments: the VAS (range: 0–10) to assess subjective pain intensity; the HADS, including subscales for anxiety (HADS-A) and depression (HADS-D); and the PSQI, covering seven domains such as sleep latency, duration, efficiency, and disturbances. Additionally, nursing adherence and satisfaction were evaluated through questionnaires. Adherence was categorized as full (100%), partial (50%), or non-adherence, while satisfaction was rated as satisfied, neutral, or dissatisfied. To minimize potential bias, all outcome assessments were performed by trained evaluators who were blinded to group allocation and intervention details.

#### 2.5.1 Minimal clinically important difference (MCID) and clinical interpretation

To enhance the clinical interpretability of outcome measures, the minimal clinically important difference (MCID) of each scale was considered when evaluating changes in pain, emotional status, and sleep quality. Based on previous literature, the MCID for the Visual Analog Scale (VAS) in neuropathic pain is approximately 1.0–1.5 points; for the Hospital Anxiety and Depression Scale, the MCID is 1.5–1.7 points for both HADS-A and HADS-D; and for the Pittsburgh Sleep Quality Index (PSQI), clinically meaningful improvement is generally defined as a reduction of approximately three points. These MCID thresholds were used as supporting criteria to determine whether statistically significant changes also represented meaningful clinical improvement.

#### 2.5.2 Psychiatric screening considerations

The Hospital Anxiety and Depression Scale (HADS) was selected as the psychological assessment tool due to its strong reliability in medical populations and its advantage of minimizing confounding by somatic symptoms that commonly overlap with chronic pain disorders. However, HADS functions as a screening rather than diagnostic instrument. No structured psychiatric interviews (e.g., DSM-5–based clinical interviews) were performed, and thus the presence of pre-existing psychiatric disorders could not be fully excluded. The interpretation of emotional outcomes is therefore limited to changes in symptom severity rather than diagnostic remission.

### 2.6 Randomization and blinding procedures

A computer-generated random number table was used to produce the allocation sequence by an independent researcher who was not involved in participant recruitment or outcome assessment. To ensure allocation concealment, group assignments were placed in sealed, opaque, sequentially numbered envelopes, which were opened only after a participant completed baseline assessment.

To minimize potential assessment bias, outcome assessors were blinded to group assignments throughout the study. The nursing staff responsible for delivering the individualized intervention did not participate in data collection, and all post-intervention evaluations (HADS-A/D, PSQI, VAS) were conducted by trained assessors who were unaware of participant group allocation.

### 2.7 Statistical analysis

All data were analyzed using SPSS version 26.0 (IBM Corporation, Armonk, NY, USA). Normally distributed continuous variables were expressed as mean ± standard deviation (*x̄*±s) and compared using independent samples *t-*tests. Non-normally distributed variables were reported as medians with interquartile ranges and analyzed using the Mann–Whitney U test. Within-group comparisons before and after intervention were conducted using the Wilcoxon signed-rank test. Categorical data were presented as frequencies and percentages and compared using the chi-square (*χ*^2^) test or Fisher’s exact test, as appropriate. All tests were two-tailed, and a *p*-value of <0.05 was considered statistically significant.

## 3 Results

### 3.1 Potential risk factors affecting pain perception in patients with TN

A total of 162 TN patients were divided into a low-VAS group (VAS = 0, *n* = 56) and a high-VAS group (VAS > 0, *n* = 106). Most demographic and general clinical variables—including marital status, occupation, education level, age, BMI, disease duration, pain location, pain type, and pain triggers—showed no significant differences between the two groups (all *p* > 0.05), indicating that baseline demographic factors were not the main contributors to pain perception.

**Figure 1:**
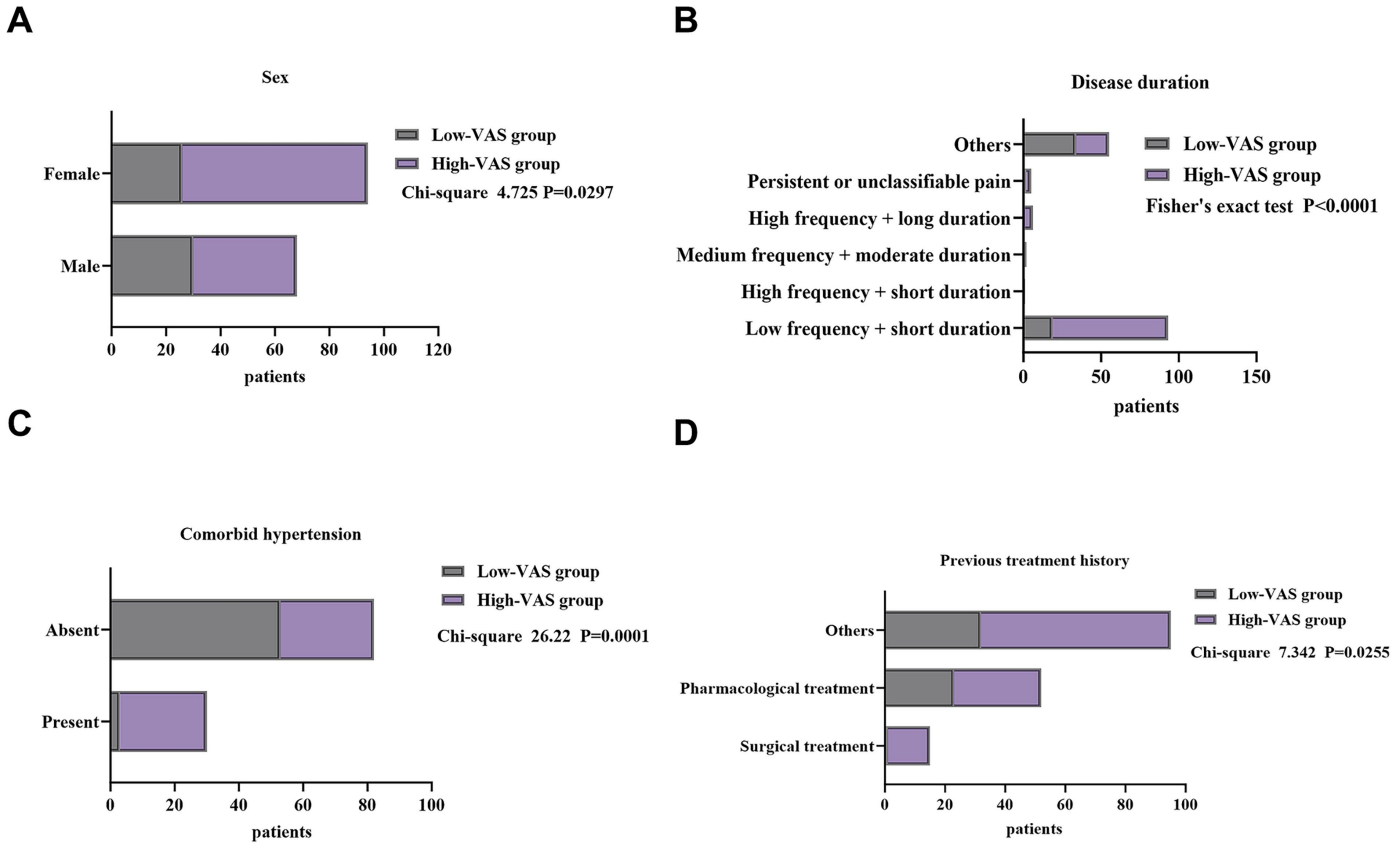
Comparison of major factors associated with pain perception between the low-VAS and high-VAS groups in patients with trigeminal neuralgia. **(A)** Sex distribution. **(B)** Pain frequency and duration patterns. **(C)** Comorbid hypertension. **(D)** Previous treatment history.

In contrast, several variables demonstrated clear and statistically significant differences. Sex distribution differed significantly (*χ*^2^ = 4.725, *p* = 0.030), with female patients more likely to be in the high-VAS group, suggesting a higher tendency toward pain perception or reporting. Pain attack patterns also differed significantly (*χ*^2^ = 28.775, *p* < 0.001). The high-VAS group predominantly experienced typical low-frequency + short-duration attacks, whereas the low-VAS group more frequently reported atypical or unclassifiable pain patterns. Comorbidities also showed differences: hypertension was significantly more common in the high-VAS group (25.47% vs. 5.36%, *χ*^2^ = 9.825, *p* = 0.002), suggesting a potential link between vascular comorbidity and higher subjective pain. Regarding treatment history, previous surgical treatment was more frequent in the high-VAS group (13.21% vs. 1.79%, *χ*^2^ = 7.342, *p* = 0.025, Table 1 and Figure 1), reflecting that patients with more severe or persistent disease tended to report higher pain levels.

**Table 1:** Potential risk factors affecting pain status in patients with TN [*x̄*±s, M (Q_25_, Q_75_), *n* (%)].

Variables		Low-VAS group (*n* = 56)	High-VAS group (*n* = 106)	t/χ^2^/Z	*p*-value
Marital status	Married	52 (92.86%)	103 (97.17%)	1.648	0.199
	Widowed	4 (7.14%)	3 (2.83%)		
Occupation	Cadre	1 (1.79%)	1 (0.94%)	9.299	0.504
	Self-employed	1 (1.79%)	3 (2.83%)		
	Worker	7 (12.5%)	13 (12.26%)		
	Technician	2 (3.57%)	0 (0.0%)		
	Teacher	0 (0.0%)	3 (2.83%)		
	Retired (active)	6 (10.71%)	16 (15.09%)		
	Retired	6 (10.71%)	8 (7.55%)		
	Unemployed	12	16 (15.09%)		
	Farming	18	38 (35.85%)		
	Clerk/Staff	3 (5.36%)	5 (4.72%)		
	Freelancer	0 (0.0%)	3 (2.83%)		
Education level	Bachelor’s degree	1 (1.79%)	3 (2.83%)	4.851	0.563
	Junior high school	15 (26.79%)	33 (31.13%)		
	Associate degree	1 (1.79%)	5 (4.72%)		
	Senior high school	5 (8.93%)	16 (15.09%)		
	Illiterate	8 (14.29%)	16 (15.09%)		
	Primary school	25 (44.64%)	31 (29.25%)		
	Technical secondary school	1 (1.79%)	2 (1.89%)		
Age		64.38 ± 10.23	61.58 ± 10.51	1.627	0.106
BMI		23.41 (21.54, 25.76)	24.26 (22.39, 27.58)	1.210	0.226
Disease duration	>2 years	27 (48.21%)	57 (53.77%)	0.454	0.501
	≤2 years	29 (51.79%)	49 (46.23%)		
Affected Branch of Trigeminal Nerve	Ophthalmic branch (V1) involvement	1 (1.79%)	4 (3.77%)	0.726	0.971
	Maxillary branch (V2) involvement	16 (28.57%)	31 (29.25%)		
	Mandibular branch (V3) involvement	16 (28.57%)	31 (29.25%)		
	Two or more branches/bilateral involvement	20 (35.71%)	36 (33.96%)		
	No clear information	3 (5.36%)	4 (3.77%)		
Pain characteristics	Electric shock-like pain	53 (94.64%)	97 (91.51%)	3.398	0.494
	Needle-prick-like pain	1 (1.79%)	4 (3.77%)		
	Cutting/tearing-like pain	0 (0.0%)	1 (0.94%)		
	Twitch-related pain	1 (1.79%)	0 (0.0%)		
	Other/unclassifiable pain	1 (1.79%)	4 (3.77%)		
Pain triggers	With clear triggers	36 (64.29%)	68 (64.15%)	1.089	0.580
	Without obvious triggers	20 (35.71%)	36 (33.96%)		
	Other triggers	0 (0.0%)	2 (1.89%)		
Comorbid diabetes	Present	1 (1.79%)	7 (6.6%)	1.812	0.178
	Absent	55 (98.21%)	99 (93.4%)		

In summary, subjective pain perception in TN patients was primarily associated with sex, hypertension, attack frequency and duration, and previous surgical history, whereas most demographic and general clinical variables showed no significant impact on VAS group classification.

### 3.2 Potential risk factors affecting psychological status in TN patients

**Figure 2:**
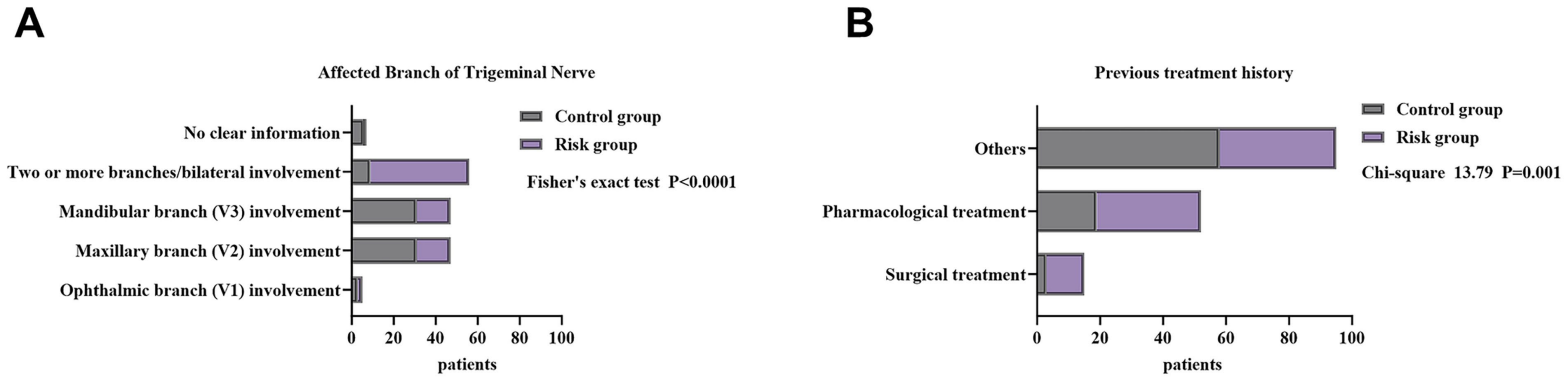
Factors associated with psychological status in patients with trigeminal neuralgia. **(A)** Distribution of trigeminal nerve involvement. **(B)** Previous treatment history.

Based on HADS-A and HADS-D scores, patients were divided into a control group (*n* = 80) and a risk group (*n* = 82). As shown in Table 2 and Figure 2, most demographic and general clinical characteristics—including sex, marital status, occupation, education level, age, BMI, disease duration, pain frequency, pain characteristics, pain triggers, and comorbidities—did not differ significantly between groups (all *p* > 0.05), indicating that baseline demographic or clinical factors were not major determinants of psychological status in TN.

**Table 2:** Potential risk factors affecting psychological status in TN patients [*x̄*±s, M (Q_25_, Q_75_), *n* (%)].

Variables		Control group (*n* = 82)	Risk group (*n* = 82)	t/χ^2^/Z	*p*-value
Sex	Male	34 (42.5%)	34 (41.46%)	0.018	0.894
	Female	46 (57.5%)	48 (58.54%)		
Marital status	Married	77 (96.25%)	78 (95.12%)	0.125	0.724
	Widowed	3 (3.75%)	4 (4.88%)		
Occupation	Cadre	2 (2.5%)	0 (0.0%)	5.427	0.861
	Self-employed	2 (2.5%)	2 (2.44%)		
	Worker	11 (13.75%)	9 (10.98%)		
	Technician	1 (1.25%)	1 (1.22%)		
	Teacher	1 (1.25%)	2 (2.44%)		
	Retired (active)	9 (11.25%)	13 (15.85%)		
	Retired	5 (6.25%)	9 (10.98%)		
	Unemployed	15 (18.75%)	13 (15.85%)		
	Farming	27 (33.75%)	29 (35.37%)		
	Clerk/Staff	5 (6.25%)	3 (3.66%)		
	Freelancer	2 (2.5%)	1 (1.22%)		
Education level	Bachelor’s degree	1 (1.25%)	3 (3.66%)	4.631	0.620
	Junior high school	25 (31.25%)	23 (28.05%)		
	Associate degree	4 (5.0%)	2 (2.44%)		
	Senior high school	7 (8.75%)	1417.07%		
	Illiterate	13 (16.25%)	11 (13.41%)		
	Primary school	29 (36.25%)	27 (32.93%)		
	Technical secondary school	1 (1.25%)	2 (2.44%)		
Age		59.5 (54, 69)	66 (57.25, 71)	1.676	0.094
BMI		23.85 (21.81, 27.52)	24.16 (22.04, 26.15)	0.082	0.935
Disease duration	>2 years	41 (51.25%)	43 (52.44%)	0.023	0.880
	≤2 years	39 (48.75%)	39 (47.56%)		
Disease duration	Low frequency + short duration	48 (60.0%)	45 (54.88%)	1.957	0.855
	High frequency + short duration	0 (0.0%)	1 (1.22%)		
	Medium frequency + moderate duration	1 (1.25%)	1 (1.22%)		
	High frequency + long duration	2 (2.5%)	4 (4.88%)		
	Persistent or unclassifiable pain	2 (2.5%)	3 (3.66%)		
	Others	27 (33.75%)	28 (34.15%)		
Pain characteristics	Electric shock-like pain	78 (97.5%)	72 (87.8%)	7.416	0.115
	Needle-prick-like pain	0 (0.0%)	5 (6.1%)		
	Cutting/tearing-like pain	0 (0.0%)	1 (1.22%)		
	Twitch-related pain	0 (0.0%)	1 (1.22%)		
	Other/unclassifiable pain	2 (2.5%)	3 (3.66%)		
Pain triggers	With clear triggers	55 (68.75%)	49 (59.76%)	1.465	0.481
	Without obvious triggers	24 (30.0%)	32 (39.02%)		
	Other triggers	1 (1.25%)	1 (1.22%)		
Comorbid hypertension	Present	12 (15.0%)	18 (21.95%)	1.297	0.255
	Absent	68 (85.0%)	64 (78.05%)		
Comorbid diabetes	Present	4 (5.0%)	4 (4.88%)	0.001	0.971
	Absent	76 (95.0%)	78 (95.12%)		

In contrast, two disease-related variables showed clear and statistically significant differences. Pain distribution differed markedly between groups (*χ*^2^ = 39.113, *p* < 0.001). The risk group had a substantially higher proportion of multiple-branch or bilateral trigeminal involvement (57.32%), whereas the control group predominantly exhibited isolated V2 or V3 involvement. This suggests that wider nerve involvement is closely associated with anxiety and depression. Previous treatment history also differed significantly (*χ*^2^ = 13.789, *p* = 0.001). The risk group showed higher proportions of patients who had undergone surgery (14.63% vs. 3.75%) or pharmacological treatment (40.24% vs. 23.75%), implying that patients with more severe or persistent disease requiring more extensive interventions were more likely to present with elevated HADS scores.

In summary, psychological distress in TN patients was primarily associated with disease severity–related features, particularly broader trigeminal involvement and more intensive treatment history, rather than with demographic or general clinical variables.

### 3.3 Potential risk factors affecting sleep quality in TN patients

**Figure 3:**
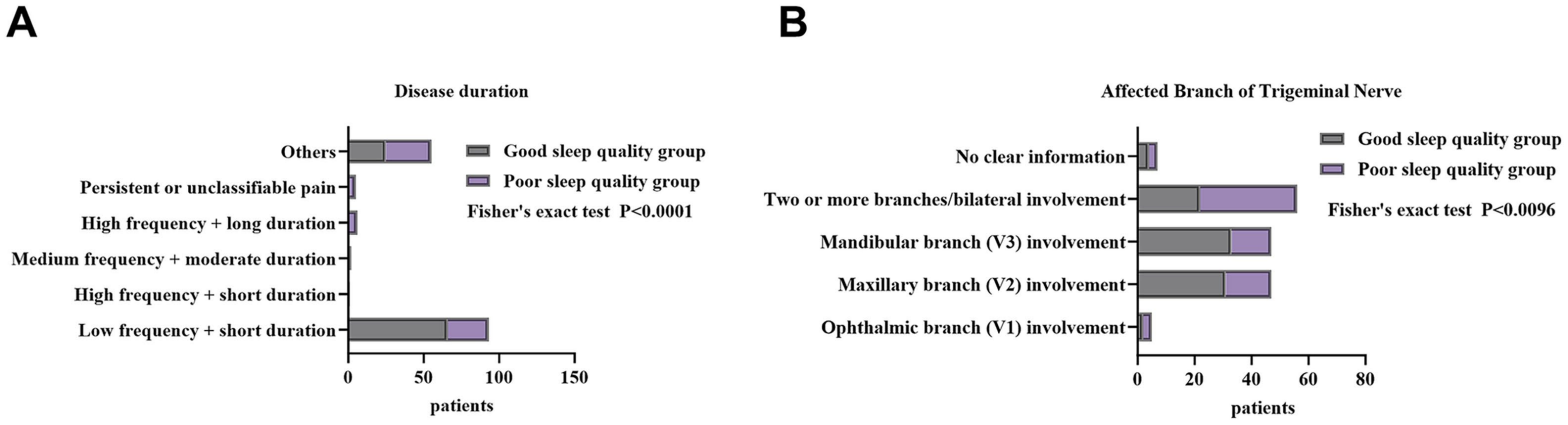
Pain-related factors associated with sleep quality in patients with trigeminal neuralgia. **(A)** Distribution of pain frequency and duration. **(B)** Distribution of trigeminal nerve involvement.

Patients were divided into a good sleep quality group (PSQI ≤ 5, *n* = 92) and a poor sleep quality group (PSQI > 5, *n* = 70). As shown in Table 3 and Figure 3, most demographic and general clinical characteristics did not differ significantly between the two groups, indicating that sleep quality in TN patients was not strongly influenced by baseline demographic factors.

**Table 3:** Potential risk factors affecting sleep quality in patients with TN [*x̄*±s, M (Q_25_, Q_75_), *n* (%)].

Variables		Good sleep quality group (*n* = 92)	Poor sleep quality group (*n* = 70)	t/χ^2^/Z	*p-*value
Sex	Male	39 (42.39%)	29 (41.43%)	0.015	0.902
	Female	53 (57.61%)	41 (58.57%)		
Marital status	Married	89 (96.74%)	66 (94.29%)	0.579	0.447
	Widowed	3 (3.26%)	4 (5.71%)		
Occupation	Cadre	2 (2.17%)	0 (0.0%)	7.790	0.649
	Self-employed	1 (1.09%)	3 (4.29%)		
	Worker	10 (10.87%)	10 (14.29%)		
	Technician	2 (2.17%)	0 (0.0%)		
	Teacher	2 (2.17%)	1 (1.43%)		
	Retired (active)	10 (10.87%)	12 (17.14%)		
	Retired	7 (7.61%)	7 (10.0%)		
	Unemployed	17 (18.48%)	11 (15.71%)		
	Farming	35 (38.04%)	21 (30.0%)		
	Clerk/Staff	4 (4.35%)	4 (5.71%)		
	Freelancer	2 (2.17%)	1 (1.43%)		
Education level	Bachelor’s degree	2 (2.17%)	2 (2.86%)	4.100	0.663
	Junior high school	29 (31.52%)	19 (27.14%)		
	Associate degree	2 (2.17%)	4 (5.71%)		
	Senior high school	13 (14.13%)	8 (11.43%)		
	Illiterate	16 (17.39%)	8 (11.43%)		
	Primary school	29 (31.52%)	27 (38.57%)		
	Technical secondary school	1 (1.09%)	2 (2.86%)		
Age		63.20 ± 9.70	61.69 ± 11.41	0.071	0.943
BMI		24.07 (21.93, 26.99)	24.22 (21.93, 27.34)	0.909	0.365
Disease duration	>2 years	51 (55.43%)	33 (47.14%)	1.095	0.295
	≤2 years	41 (44.57%)	37 (52.86%)		
Pain characteristics	Electric shock-like pain	88 (95.65%)	62 (88.57%)	5.623	0.229
	Needle-prick-like pain	1 (1.09%)	4 (5.71%)		
	Cutting/tearing-like pain	0 (0.0%)	1 (1.43%)		
	Twitch-related pain	0 (0.0%)	1 (1.43%)		
	Other/unclassifiable pain	3 (3.26%)	2 (2.86%)		
Pain triggers	With clear triggers	61 (66.3%)	43 (61.43%)	0.421	0.810
	Without obvious triggers	30 (32.61%)	26 (37.14%)		
	Other triggers	1 (1.09%)	1 (1.43%)		
Comorbid hypertension	Present	16 (17.39%)	14 (20.0%)	0.179	0.672
	Absent	76 (82.61%)	56 (80.0%)		
Comorbid diabetes	Present	4 (4.35%)	4 (5.71%)	0.158	0.727
	Absent	88 (95.65%)	66 (94.29%)		
Previous treatment history	Surgical treatment	6 (6.52%)	9 (12.86%)	1.921	0.383
	Pharmacological treatment	30 (32.61%)	22 (31.43%)		
	Others	56 (60.87%)	39 (55.71%)		

In contrast, several pain-related variables showed clear differences. Pain frequency and duration were significantly associated with sleep quality (*χ*^2^ = 25.084, *p* < 0.001). The good sleep group predominantly exhibited low-frequency + short-duration attacks (71.74%), while the poor sleep group had substantially higher proportions of high-frequency, prolonged, or persistent pain patterns, suggesting that more severe pain episodes contribute to sleep disturbance. Pain distribution also differed significantly (*χ*^2^ = 12.628, *p* = 0.010). Nearly half of the poor sleep group (48.57%) had multiple-branch or bilateral trigeminal involvement, compared with 23.91% in the good sleep group. This indicates that broader nerve involvement is closely linked to worse sleep quality. Other variables—including pain characteristics, pain triggers, comorbidities, and previous treatment history—showed no statistically significant differences between groups.

In summary, sleep quality in TN patients was mainly affected by disease severity–related factors, particularly more frequent and longer-lasting pain episodes and wider nerve involvement, rather than by demographic or general clinical characteristics.

### 3.4 Baseline characteristics of the two groups

A total of 64 TN patients were randomly assigned into a control group (*n* = 32) and an intervention group (*n* = 32) using a random number table. Comparisons of baseline demographic characteristics, as well as psychological and physiological indicators, are summarized below:

There were no significant differences in age (64.34 ± 9.29 vs. 61.13 ± 7.07 years; *t* = 1.56, *p* = 0.124) or BMI [median (IQR): 20.72 (19.69, 23.07) vs. 21.29 (19.85, 25.39); *Z* = 0.947, *p* = 0.344]. Gender distribution was also similar, with 40.63% males in the control group and 46.88% in the intervention group (*χ*^2^ = 0.254, *p* = 0.614). Marital status was predominantly “married” in both groups (87.5% vs. 78.13%), with no significant difference (*χ*^2^ = 0.988, *p* = 0.320). A disease duration >2 years was observed in 28.13% of the control group and 21.88% of the intervention group (*χ*^2^ = 0.333, *p* = 0.564), with no statistical significance. Regarding psychological and sleep status, the median HADS-A score was 5 (3, 6.75) in the control group and 5 (4, 7) in the intervention group (*Z* = 0.917, *p* = 0.359), while median HADS-D scores were both 5 (with minor IQR differences; *Z* = −0.286, *p* = 0.775). Median PSQI scores were 5 (2.25, 6) in the control group and 4.5 (3.25, 6) in the intervention group (*Z* = −0.075, *p* = 0.940), indicating comparable baseline sleep quality. Median VAS scores were slightly lower in the intervention group [3 (2, 5) vs. 4 (3, 5)], though the difference was not statistically significant (*Z* = −1.816, *p* = 0.069) (Table 4).

**Table 4:** Comparison of baseline characteristics between patients receiving different nursing interventions.

Variables		Control group (*n* = 32)	Intervention group (*n* = 32)	t/χ^2^/Z	*p-*value
Age		64.34 ± 9.29	61.13 ± 7.07	1.56	0.124
BMI		20.72 (19.69, 23.07)	21.29 (19.85, 25.39)	0.947	0.344
Sex	Male	13 (40.63%)	15 (46.88%)	0.254	0.614
	Female	19 (59.38%)	17 (53.13%)		
Marital status	Married	28 (87.5%)	25 (78.13%)	0.988	0.320
	Widowed	4 (12.5%)	7 (21.88%)		
Disease duration	>2 years	9 (28.13%)	7 (21.88%)	0.333	0.564
	≤2 years	23 (71.88%)	25 (78.13%)		
HADS-A		5 (3, 6.75)	5 (4, 7)	0.917	0.359
HADS-D		5 (3, 7)	5 (3, 7)	−0.286	0.775
PSQI		5 (2.25, 6)	4.5 (3.25, 6)	−0.075	0.94
VAS		4 (3, 5)	3 (2, 5)	−1.816	0.069

### 3.5 Comparison of psychological status improvement after intervention

**Figure 4:**
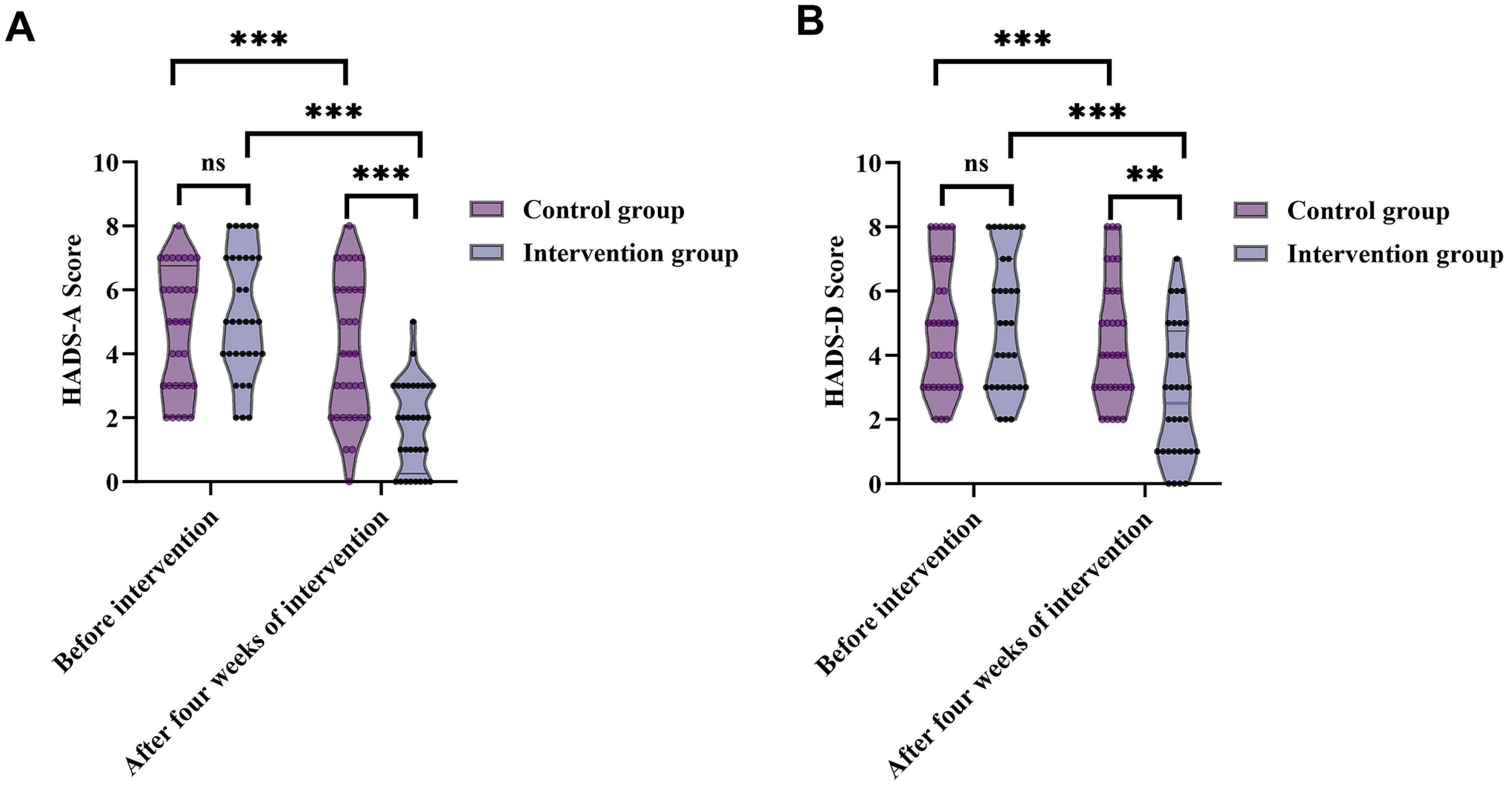
Changes in anxiety and depression levels before and after the four-week intervention in patients with trigeminal neuralgia. **(A)** HADS-A scores, after the intervention, the intervention group showed a marked reduction in HADS-A (from 95% CI: 4.52–5.91 to 95% CI: 1.26–2.24), whereas changes in the control group were minimal (from 95% CI: 4.09–5.47 to 95% CI: 3.30–4.89). **(B)** HADS-D scores also showed significant improvement in the intervention group (from 95% CI: 4.27–5.79 to 95% CI: 2.01–3.49), while the control group exhibited no meaningful change (from 95% CI: 4.15–5.60 to 95% CI: 3.75–5.13). ns, not significant; **p < 0.01; ***p < 0.001.

Although the sample size of the prospective phase was relatively modest (*n* = 64), the analyses incorporated 95% confidence intervals (CI) for all primary outcomes (VAS, HADS-A, HADS-D, and PSQI) to improve statistical rigor. The reported CIs demonstrate that the observed intergroup and intragroup differences are consistent and clinically meaningful, supporting the reliability of the findings despite the moderate sample size. As shown in Figure 4, there were no significant differences in anxiety or depression scores between the two groups before the intervention. Median HADS-A scores were 5 (3, 6.75) in the control group and 5 (4, 7) in the intervention group (Z = 0.917, *p* = 0.359) median HADS-D scores were both 5 (3, 7) (*Z* = 0.286, *p* = 0.775).

After 4 weeks of intervention, anxiety and depression levels in the intervention group declined significantly compared to the control group. The median HADS-A score dropped to 2 (0.25, 3) in the intervention group, significantly lower than 4 (2, 6) in the control group (*Z* = 3.662, *p* < 0.001); the HADS-D score fell to 2.5 (1, 4.75) in the intervention group, also significantly lower than 4 (3, 6) in the control group (*Z* = 2.576, *p* = 0.010).

Within-group comparisons showed significant post-intervention reductions in both anxiety and depression scores in both groups. In the control group, HADS-A and HADS-D scores significantly decreased (*Z* = 3.397, *p* = 0.001; *Z* = 3.742, *p* < 0.001). The intervention group showed even greater improvement (HADS-A: *Z* = 4.984, *p* < 0.001; HADS-D: *Z* = 4.950, *p* < 0.001). These results suggest that targeted nursing intervention was more effective than routine care in alleviating anxiety and depression symptoms in TN patients.

### 3.6 Sleep quality improvement after different nursing interventions

**Figure 5:**
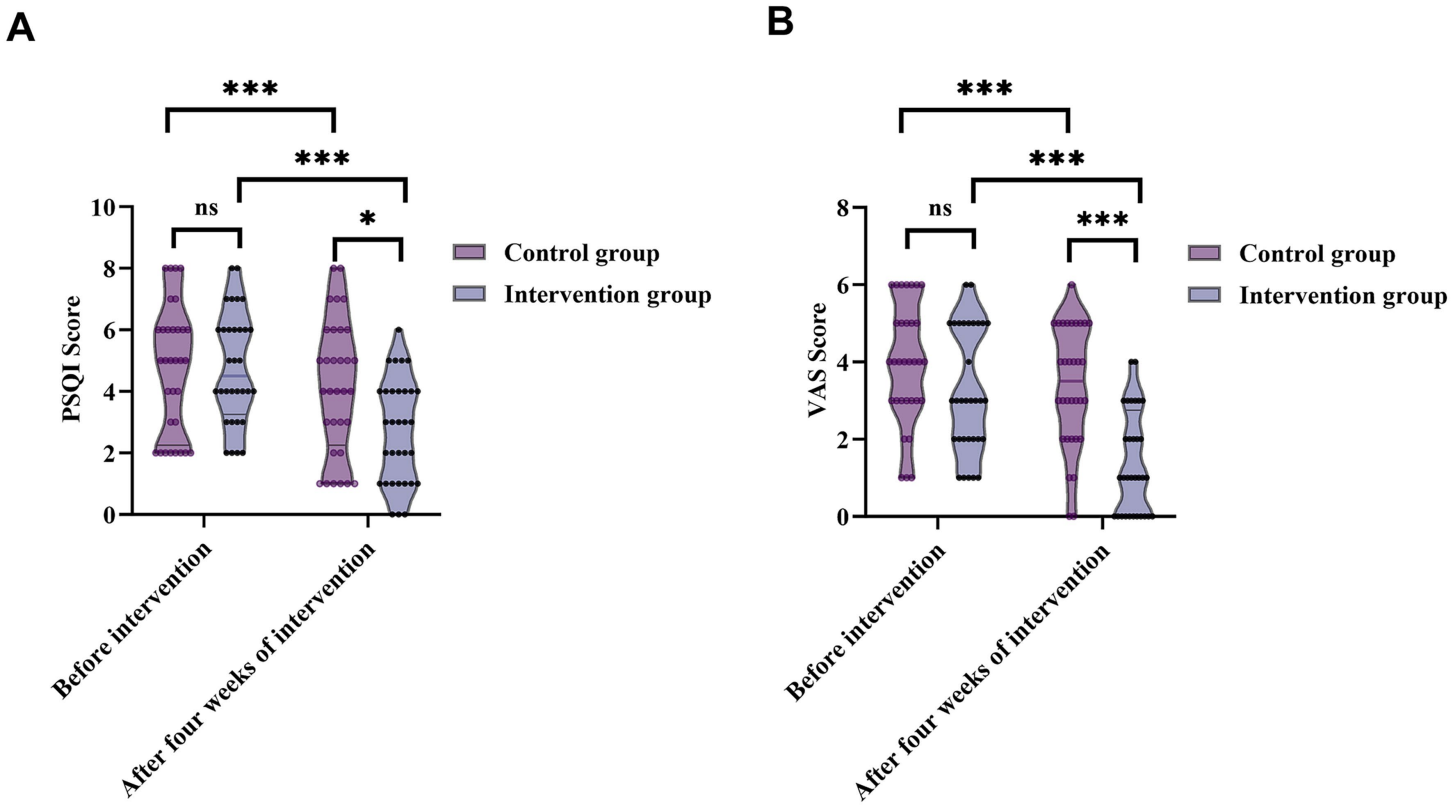
Changes in sleep quality (PSQI) and pain intensity (VAS) before and after the four-week intervention in patients with trigeminal neuralgia. **(A)** PSQI scores from 95% CI: 4.14–5.43 to 95% CI: 2.08–3.30—while the control group demonstrated only minimal improvement (from 95% CI: 4.01–5.49 to 95% CI: 3.37–4.94). **(B)** VAS pain scores from 95% CI: 2.67–3.83 at baseline to 95% CI: 0.90–1.85 after 4 weeks. In the control group, changes were small (from 95% CI: 3.41–4.53 to 95% CI: 2.80–3.95). ns, not significant; *p < 0.05; **p < 0.01; ***p < 0.001.

Prior to intervention, PSQI scores showed no significant difference between groups [control: 5 (2.25, 6); intervention: 4.5 (3.25, 6); *Z* = 0.075, *p* = 0.940], indicating similar baseline sleep quality. After 4 weeks, the intervention group’s median PSQI score significantly decreased to 3 (1, 4), compared to 4 (2.25, 6) in the control group (*Z* = 2.446, *p* = 0.014) (Figure 5A). Paired comparisons within groups also revealed significant PSQI reductions in both groups (control: *Z* = 4.359, *p* < 0.001; intervention: *Z* = 4.964, *p* < 0.001), indicating that nursing care effectively improved sleep quality, with more notable improvements in the intervention group.

### 3.7 Comparison of pain relief between the two groups

Before the intervention, no significant difference was observed in VAS scores [control: 4 (3, 5); intervention: 3 (2, 5); *Z* = 1.816, *p* = 0.069], indicating comparable baseline pain severity. After 4 weeks, VAS scores in the intervention group decreased markedly to 1 (0, 2.75), significantly lower than 3.5 (2, 5) in the control group (*Z* = 3.652, *p* < 0.001). Within-group analyses showed significant reductions in VAS scores in both groups (control: *Z* = 4.359, *p* < 0.001; intervention: *Z* = 4.933, *p* < 0.001) (Figure 5B), demonstrating that nursing interventions effectively alleviated TN-related pain, with superior outcomes in the intervention group.

### 3.8 Patient adherence and satisfaction with nursing care

In terms of satisfaction, the total satisfaction rate in the intervention group was 93.75%, notably higher than 84.37% in the control group. Among control patients, 17 (53.13%) reported full satisfaction, 10 (31.25%) partial satisfaction, and 5 (15.63%) dissatisfaction. In the intervention group, 27 (84.38%) were fully satisfied, 3 (9.38%) partially satisfied, and 2 (6.25%) dissatisfied. This difference was statistically significant (*χ*^2^ = 7.328, *p* = 0.035), suggesting that targeted nursing intervention also significantly enhanced patient satisfaction. The overall adherence rate in the intervention group was 96.87%, significantly higher than 62.5% in the control group. Specifically, in the control group, 18 patients (56.25%) were fully adherent, 2 (6.25%) partially adherent, and 12 (37.5%) nonadherent. In the intervention group, 26 patients (81.25%) were fully adherent, 5 (15.63%) partially adherent, and only 1 (3.13%) nonadherent. Chi-square test results showed a significant group difference in adherence (*χ*^2^ = 12.048, *p* = 0.001), indicating that targeted nursing interventions significantly improved adherence in TN patients (Table 5).

**Table 5:** Comparison of patient satisfaction and treatment adherence between the two groups.

Groups	Control group (*n* = 32)	Intervention group (*n* = 32)	*χ* ^2^	*p*
100% satisfaction	17 (53.13%)	27 (84.38%)	7.328	0.035
50% satisfaction	10 (31.25%)	3 (9.38%)		
Dissatisfaction	5 (15.63%)	2 (6.25%)		
100% adherence	18 (56.25%)	26 (81.25%)	12.048	0.001
50% adherence	2 (6.25%)	5 (15.63%)		
Nonadherence	12 (37.5%)	1 (3.13%)		

## 4 Discussion

Trigeminal neuralgia (TN), a neuropathic pain disorder that severely affects quality of life, has traditionally been studied from the perspectives of etiology, imaging localization, and surgical management (18). However, despite advances in neuromodulation techniques such as microvascular decompression and radiofrequency ablation (19), improvements in functional recovery and psychosocial outcomes remain limited (20). Existing literature focuses largely on pain relief and recurrence rates, whereas psychological distress and sleep disturbances—highly prevalent among TN patients (21)—are often insufficiently recognized and inadequately managed in routine care.

Recent studies increasingly suggest a strong interplay between chronic pain, emotional state, and sleep quality, yet three major limitations persist in the current evidence base: 1. Cross-sectional designs dominate, preventing causal inference. 2. Pain, psychological symptoms, and sleep problems are often evaluated in isolation, lacking an integrated framework. 3. Stratified or individualized nursing protocols based on patient characteristics remain scarce, resulting in nonspecific and fragmented clinical management. To fill these gaps, our study adopted a two-stage design. The retrospective analysis first clarified the relationships between pain characteristics and psychosocial status, while the prospective randomized controlled trial (RCT) evaluated the clinical utility of individualized comprehensive nursing interventions.

### 4.1 Associations between pain characteristics and psychosocial burden

Our retrospective findings revealed that attack frequency, distribution of trigeminal nerve involvement, and previous treatment history were strongly associated with pain severity, emotional distress, and sleep impairment. Patients with multi-branch or bilateral involvement exhibited substantially higher anxiety and depression scores, consistent with prior evidence (22). Frequent or prolonged attacks and persistent atypical pain further contributed to worse sleep quality. Sex differences were also noted: female patients tended to report higher pain burden, echoing findings from De Stefano et al. (23) and Liu’s et al. (24) observation that wider pain distribution is more likely to affect emotional state. Comorbid hypertension appeared related to higher pain perception, aligning with studies linking vascular comorbidities to pain sensitivity. These findings highlight that TN is not a homogeneous condition; rather, patients display distinct symptom clusters that warrant personalized management strategies.

### 4.2 Need for stratified and targeted nursing management

Although individualized nursing concepts have been explored in some chronic disease contexts, their application in TN remains limited and insufficiently standardized. Our data reinforce the necessity of stratifying patients based on pain patterns, emotional vulnerability, and sleep problems, thereby establishing a more precise and continuous nursing model. By integrating multidimensional assessment—pain intensity, psychological status, sleep quality, and clinical history—this study contributes a more comprehensive framework for TN nursing research.

### 4.3 Prospective randomized controlled trial: validation of individualized nursing

The second stage of the study evaluated the clinical benefits of individualized nursing interventions using a prospective RCT. The individualized care model significantly improved: pain intensity (VAS), anxiety and depression (HADS-A/D) (25), sleep quality (PSQI), treatment adherence and patient satisfaction. These results not only demonstrate the effectiveness of tailored nursing interventions but also provide causal evidence supporting the implementation of stratified nursing strategies, addressing an important gap previously highlighted in the literature.

### 4.4 Clinical implications

The integrated findings from both study stages confirm that pain characteristics are closely linked to emotional state and sleep quality, and that addressing these domains through individualized nursing can significantly enhance clinical outcomes. This two-step approach—risk-factor identification followed by targeted intervention—offers a feasible, evidence-based framework for optimizing TN management.

### 4.5 Clinical significance based on MCID

Although the prospective intervention yielded statistically significant improvements in pain intensity, anxiety, depression, and sleep quality, the magnitude of change also exceeded the established MCID thresholds for all scales, indicating that the improvements were not only statistically detectable but also clinically meaningful. Specifically, reductions in VAS exceeded the 1.0–1.5–point threshold for clinically relevant pain improvement, while decreases in HADS-A and HADS-D surpassed their respective 1.5–1.7–point MCID values. Similarly, the reduction in PSQI scores in the intervention group approached or exceeded the approximately 3–point threshold considered meaningful for sleep improvement. These findings support the practical relevance of individualized nursing interventions in managing multidimensional symptom burdens in TN patients.

### 4.6 Limitations and future directions

Despite its theoretical and empirical strengths, this study has several limitations. First, the sample size in the prospective phase was relatively small, and the follow-up duration was short, limiting the evaluation of long-term intervention effects. Second, the assessment of psychological and sleep status relied mainly on self-reported scales, without objective physiological indicators (e.g., heart rate variability, sleep structure analysis) to support the findings, which may introduce reporting bias. Finally, as a single-center study, the implementation of nursing interventions relied on a specific team, and generalizability should be validated through multi-center studies. Additionally, the sample size of the prospective phase (*n* = 64), although based on an *a priori* power calculation, is relatively small and may limit the statistical power for multidimensional outcomes. Larger multicenter studies are therefore required to confirm and extend our results. Despite these strengths, several limitations warrant attention. Psychological assessment relied solely on the HADS, which—while widely validated for use in medical settings—cannot replace formal psychiatric diagnostic procedures. The absence of structured diagnostic interviews (e.g., DSM-5–based assessments) means that pre-existing anxiety or depressive disorders could not be fully excluded. Therefore, the observed improvements should be interpreted as reductions in symptom severity, rather than definitive changes in diagnostic status. Future studies incorporating standardized psychiatric interviews or multidisciplinary psychological evaluation may provide a more comprehensive understanding of the emotional burden associated with trigeminal neuralgia and the mechanisms through which nursing interventions exert their effects.

In summary, this study systematically identified and analyzed the triad of pain, emotional state, and sleep status in patients with TN, and developed a risk factor–oriented individualized nursing intervention pathway. The intervention was found to be effective in alleviating pain, improving mood, and enhancing sleep quality. These results not only provide evidence-based support for TN care but also lay a theoretical foundation for future development of nursing intervention strategies based on the interactive “pain-emotion-behavior” model. Future studies should further explore the generalizability of this intervention pathway through multi-center, large-sample, and long-term follow-up designs, and investigate additional nursing models based on both physiological and psychological mechanisms.

## 5 Conclusion

This study provides an integrated model for assessing pain-related suffering in patients with trigeminal neuralgia by examining the combined influence of pain characteristics, psychological distress, and sleep disturbance. Through a two-stage design, we identified key symptom patterns associated with higher disease burden and demonstrated, via a prospective randomized controlled trial, that individualized nursing interventions can meaningfully improve emotional status, sleep quality, and pain intensity.

Although the findings offer a promising framework for stratified and patient-centered nursing care, the results are derived from a single-center cohort and a relatively short intervention period; therefore, the conclusions are not immediately generalizable. Nevertheless, the present work highlights a feasible direction for future research. Larger multicenter studies, longer follow-up durations, and integration of neuroimaging or physiological markers may further refine stratified management strategies and validate the long-term benefits of individualized nursing for trigeminal neuralgia.

## Data Availability

The original contributions presented in the study are included in the article/supplementary material, further inquiries can be directed to the corresponding author.
